# Chronic Stroke Survivor Engaging in Balance Training on a Dual-Belt Treadmill Using Open-Skills Learning: A Case Report

**DOI:** 10.7759/cureus.77390

**Published:** 2025-01-13

**Authors:** Hiroki Yokoyama, Masanori Wakida, Hisayuki Ogura, Kimitaka Hase

**Affiliations:** 1 Rehabilitation, Kansai Medical University Kuzuha Hospital, Hirakatashi, JPN; 2 Faculty of Rehabilitation, Kansai Medical University, Hirakata City, JPN; 3 Department of Physical Medicine and Rehabilitation, Kansai Medical University Hospital, Hirakata City, JPN

**Keywords:** balance, case report, fall prevention, rehabilitation, stroke

## Abstract

Balance impairment after a stroke is a common impediment to activities of daily living and can lead to falls. Balance ability is particularly associated with social participation; therefore, targeted rehabilitation to prevent falls is crucial. We have previously developed a dual-belt treadmill task for repetitive multidirectional stepping based on open-skill training, which demonstrated significant efficacy in balance training for older adults. However, the effect on stroke survivors, who are more likely to move in a manner that compensates for their non-paretic limbs, remains unclear. This report presents the specific application of the dual-belt treadmill task in a chronic stroke survivor who experienced falls outdoors. The participant was a woman in her 60s with left hemiplegia, two years post-stroke, who walked independently. She attended a day rehabilitation program twice a week, receiving 40-minute sessions of exercise therapy. However, after experiencing two falls, a dual-belt treadmill intervention was introduced. This report examines the changes in physical function and fall-related outcomes over two three-month periods before and after the introduction of the dual-belt treadmill, using an AB design. Following the dual-belt treadmill intervention, a reduction in the number of falls was observed, along with increased muscle strength in the affected limb. The dual-belt treadmill intervention may be effective in reducing falls in chronic stroke patients.

## Introduction

Stroke can lead to motor paralysis, sensory deficits, muscle weakness, and abnormal muscle tone [1-3], which can potentially cause various difficulties in daily life and social participation. Balance impairment is one of the major barriers to community mobility among stroke patients [4]. Studies have shown that impaired balance is a risk factor for falls among community-dwelling stroke survivors and is associated with a fear of falling [5,6]. Consequently, maintaining adequate balance function plays a crucial role in fall prevention during outdoor activities. However, despite various balance-focused interventions, including the use of movable platforms, treadmills, and manual perturbation techniques delivered by physical therapists, evidence regarding effective interventions to reduce falls in stroke survivors remains insufficient [7]. One challenge may be that stroke survivors are more likely to move in a manner that compensates with the non-paretic lower limb, which may prevent them from fully engaging balance control in the paretic limb when responding to perturbation stimuli.

To improve balance abilities, we developed a dual-belt treadmill (DBT) training based on open-skill learning principles. Briefly, the DBT consists of a treadmill with two separate belts. It is equipped with handrails on the front and sides, and four touch buttons located on the front, back, left, and right handrails. While walking on the treadmill, participants must shift between lanes to press buttons in a sequence corresponding to numbers that are randomly displayed on a monitor in front of them. We reported that interventions using the DBT showed improvement in dynamic balance in older adults, as measured by the Community Balance and Mobility Scale [8].

The key feature of DBT is its promotion of frequent multidirectional stepping movements based on open-skill learning. Motor skills are categorized as either open or closed skills; open-skill learning occurs in dynamic, changing environments, while closed-skill learning happens in predictable, static ones [9]. Open-skill learning requires participants to adapt to unpredictable stimuli through active decision-making [9]. DBT incorporates this principle to effectively train complex dynamic balance through voluntary and reactive multidirectional gait training. Since it requires frequent multidirectional stepping with both lower limbs, DBT may benefit not only older adults but also stroke patients who tend to compensate with their non-paretic limb.

Herein we showcase the effectiveness of a DBT intervention in a chronic stroke survivor with a history of recurrent falls during regular training sessions. A history of multiple falls is recognized as a significant risk factor for future falls, underscoring the need for thorough evaluation and targeted interventions [10]. We evaluated the effectiveness of a three-month DBT intervention program, administered once weekly, in reducing fall frequency through a retrospective comparison with the usual care period.

## Case presentation

Patient history

**Figure 1:**
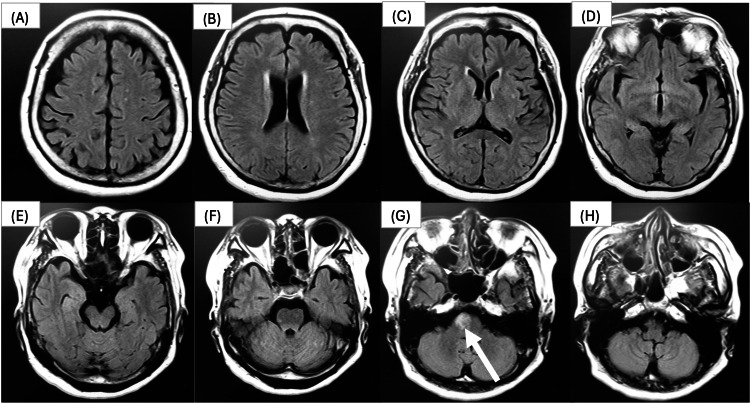
Five days after onset, T2 FLAIR image. (A, B, C, D, E, F, H) Brain images by cross-section. (G) High signal intensity observed in the pons. FLAIR: Fluid-Attenuated Inversion Recovery.

The patient was a woman in her 60s diagnosed with a right pontine infarction 2 years before and presenting with left hemiplegia of the upper and lower limbs (Figure 1).

Her medical history included a right total knee arthroplasty performed 8 years prior and a left unicompartmental knee arthroplasty performed 2 years prior. She lived with her husband and was responsible for all household chores such as laundry, cooking, cleaning, and shopping. She attended our day rehabilitation service twice a week with the aim of improving left upper limb function and enhancing her walking ability both indoors and outdoors.

The severity of paresis was classified as Brunnstrom stage IV for both upper and lower extremities. No sensory or cognitive impairments were observed. Additionally, the patient had no history of falls. She was independent in outdoor walking using an ankle foot orthosis and a cane, achieving a Functional Ambulation Categories score of 5. Her comfortable walking speed over 10 meters, using the cane and orthosis, was recorded at 0.89 m/s. Knee extension muscle strength was measured using a handheld dynamometer (μ-TasMF-01, Anima Corporation, Tokyo, Japan), yielding values of 22.1 kgf for the right side and 18.6 kgf for the left side. The Mini Mental State Examination-Japanese score was 29 points, indicating no cognitive impairment. The EQ-5D-5L, based on a 5-point scale with 1 being the highest, recorded the following scores: mobility 2 points, self-care 4 points, usual activities 3 points, pain/discomfort 2 points, and anxiety/depression 1 point. The calculated quality of life (QOL) score was 0.61527/1, in accordance with a previous study targeting Japanese individuals [11]. The details of this report were thoroughly explained to the patient both verbally and in writing, and informed consent was obtained.

Intervention overview

**Figure 2:**
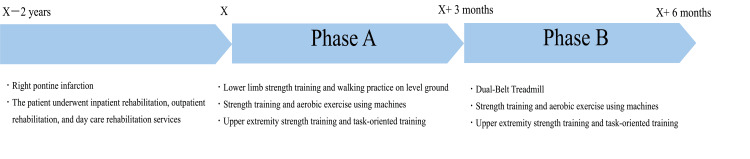
Intervention overview. The patient experienced a right pontine infarction two years ago, resulting in left hemiplegia. Following the onset, the patient underwent inpatient rehabilitation, followed by outpatient and daycare rehabilitation services. In [specific year], the intervention described in this report began. The intervention consisted of two phases, each lasting three months with twice-weekly sessions. In Phase A, one session per week focused on left upper extremity strength training and task-oriented training. The other session included 20 minutes of lower extremity strength training and gait training, plus 20 minutes of machine-based lower extremity strength training and aerobic exercise. In Phase B, one weekly session continued with left upper extremity strength training and task-oriented training, while the other session consisted of 20 minutes of dual-belt treadmill training, plus 20 minutes of machine-based lower extremity strength training and aerobic exercise.

This case report outlines two distinct phases (Figure 2) implemented using an AB design. Phase A consisted of the usual care period, during which the patient experienced repeated outdoor falls. In response to these incidents, Phase B was implemented, incorporating DBT training to specifically address balance deficits and reduce the risk of falls.

During Phase A, the patient participated in two weekday rehabilitation sessions lasting 40 minutes each; one session per week focused on task-oriented practice and strength training aimed at improving left upper limb function, while the other session focused on lower limb strength training (such as squats) and walking practice on level ground, undertaken for 3 months. Each intervention session lasted 20 minutes, and outside the intervention time, a total of 20 minutes was spent on strength training and aerobic exercise (10 minutes each) using an abduction machine (Weltonic WT-04, Minato Medical Science Co. Ltd, Osaka, Japan) and a Recumbent Cross Trainer (NuStep T4r, OG Wellness, Tokyo, Japan).

Over three months, two falls were noted during Phase A. The first fall occurred outdoors when the patient failed to step over a roadside gutter, causing her foot to catch and resulting in a fall. The second fall happened while she was moving sideways to hang laundry and lost her balance, leading to a fall. The Barthel Index, which measures independence in daily activities, was 95 points and showed no change, indicating a high level of functional ability in daily life. Her comfortable walking speed for 10 meters improved slightly from that at the initial assessment, recorded at 0.94 m/s. From this point, the Berg Balance Scale (BBS), which indicates fall risk, was used to evaluate balance, with a score of 44/56 [10]. Knee extension muscle strength was 24.1/18.2 kgf (right/left), indicating an increase in muscular capability. In the EQ-5D-5L, the scores were as follows: mobility 2 points, self-care 2 points, usual activities 3 points, pain/discomfort 2 points, and anxiety/depression 1 point, resulting in a QOL score of 0.695903/1.

The patient expressed anxiety about future falls during outdoor activities following her earlier incidents. Consequently, the DBT was proposed and initiated in Phase B. Considering that falls occurred during conventional gait and strength training sessions, we determined that training focused on advanced balance skills was necessary, particularly for improving the weight-bearing ability of the paretic limb and postural control in response to perturbations encountered during household tasks. Since DBT has been shown to improve lateral balance control ability, and one of the patient's falls occurred during lateral movement while performing household activities, it was deemed especially suitable in this case [8].

Dual-belt treadmill intervention

**Figure 3:**
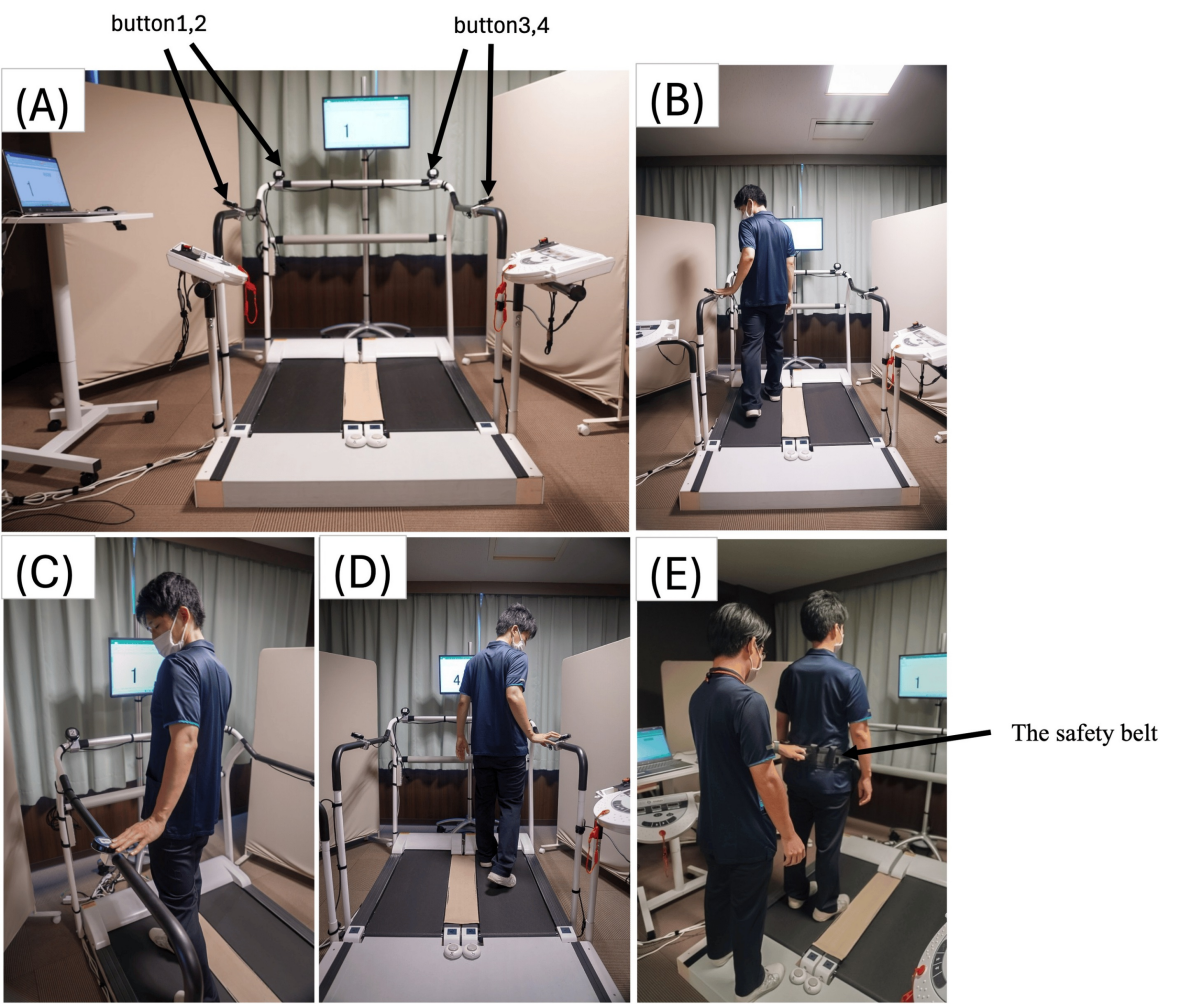
Intervention procedures. (A) The device consists of a treadmill divided into two belts. Each belt is 105 cm long and 37 cm wide, with handrails on the front and sides. The space between the two belts is 20 cm, covered by a seat to allow the subject to move between each belt. Four touch buttons are located on the front, back, left, and right handrails. (B, C, D) Training scene. The participants press the button with the same number as the number displayed on the monitor in front of them as they move from one side of the treadmill to the other. The numbers shown on the monitor are randomly displayed from numbers 1 to 4, and the numbers change randomly each time a button is pressed. (E) The subject wears a safety belt, and the physical therapist stands behind the subject. If the subject starts to lose his/her balance, the physical therapist prevents the fall by pulling on the safety belt.

Treadmill Intervention During Phase B, the patient utilized the dual-belt treadmill (Anima Corporation, Tokyo, Japan), actively switching between the left and right lanes while sequentially pressing one of four buttons corresponding to randomly displayed numbers on a monitor (Figure 3). The treadmill belts measured 105 cm in length and 37 cm in width. The patient was instructed to observe the numbers on the monitor, transition to the designated lane without stepping on the 20-cm-wide plate between the lanes, and press the button as quickly as possible with the hand corresponding to the lane. The initial walking speed was set at a comfortable level to accommodate the patient's strong anxiety about falling. One lane started at 0.38 m/s and was gradually increased to 0.47 m/s, while the other lane was set 10-20% slower. This setup aimed to provide postural control training for the lower limbs and trunk by introducing perturbations through acceleration and deceleration during transitions. Comfortable speeds were assessed in each session and gradually increased. The patient was instructed to avoid holding the handrails unless necessary for balance. An ankle foot orthosis was worn throughout the intervention, and a safety belt was used while a physical therapist stood behind the patient to prevent falls. While our previous study utilized four sets of 3-minute sessions, this protocol was adapted based on the patient's fatigue level. Each session consisted of two to three sets lasting 2-5 minutes each, with 2-3 minutes of seated rest between sets [8]. The total session time was approximately 20 minutes. Outside the intervention, as in Phase A, the patient performed a total of 20 minutes of exercise, consisting of strength training and aerobic exercise using the abduction machine (Weltonic WT-04, Minato Medical Science Co. Ltd, Osaka, Japan) and the Recumbent Cross Trainer (NuStep T4r, OG Wellness, Tokyo, Japan).

The results are summarized in Table 1. After six months, no falls were reported during Phase B. Although formal questionnaires assessing the fear of falling and balance confidence were not administered, the patient reported feeling less afraid of falling and more confident when walking outdoors. The Barthel Index remained unchanged at 95 points. Her comfortable walking speed over 10 meters was 0.93 m/s. The BBS score improved to 46/56, with gains observed in the tandem stance and single-leg stance items. Both knee extension muscle strengths increased to 26.1 kgf on the right and 23.4 kgf on the left. In the EQ-5D-5L, the patient's scores were as follows: mobility 1 point, self-care 2 points, usual activities 2 points, pain/discomfort 2 points, and anxiety/depression 2 points, resulting in a QOL score of 0.728713/1. The number of rehabilitation sessions was 18 in Phase A and 25 in Phase B, including eight sessions of DBT training during Phase B.

**Table 1:** Outcomes at various time periods. BBS: Berg Balance Scale; EQ5D-5L: EuroQol 5-Dimension 5-Level; QOL: Quality of Life; BI: Barthel Index; R/L: Right/Left; m/s: meters per second; kgf: kilogram-force; m: meters.

	first time	After the end of phase A	After the end of phase B
		(18 times attended）	(25 times attended）
The number of falls in the last three months (times)	0	2	0
Knee joint extensor strength (R/L) (kgf)	22.1/18.6	24.1/18.2	26.1/23.4
10 m walking speed (m/s)	0.89	0.94	0.93
BBS (point)	ー	44	46
EQ5D-5L QOL Score (point)	0.615	0.695	0.728
Mobility (point)	2	2	1
Self-care (point)	4	2	2
Usual activities (point)	3	3	2
Pain/Discomfort (point)	2	2	2
Anxiety/Depression (point)	1	1	2
BI (point)	95	95	95

## Discussion

This case report describes a chronic stroke survivor who experienced an outdoor fall during a 3-month period of regular rehabilitation 2 years post-stroke (Phase A) and subsequently underwent a 3-month DBT intervention (Phase B) to evaluate the effectiveness of DBT. During Phase A, usual training included upper limb function exercises, lower limb strength training using exercise equipment, and walking practice. Following multiple falls during this period, DBT training was incorporated into the regular rehabilitation regimen in Phase B. The evaluation after Phase B revealed a reduction in the number of falls, along with improvements in knee extension muscle strength in both the paretic and non-paretic lower limbs, as well as enhanced balance ability.

First, the increase in knee extension muscle strength on the paretic side was greater after the Phase B intervention compared to that in Phase A. This improvement may have been influenced by the increased active use of the paretic side required during DBT. In DBT, patients land on the paretic side while transferring on the treadmill, or they push off with the paretic side to move laterally. Such movements, particularly those involving lateral stepping, demand significant knee extension power, likely resulting in improved muscle strength of the paretic side [12]. In stroke patients, muscle strength in the paretic lower limb is more strongly associated with mobility functions compared to that in the non-paretic limb [13]. During Phase A, conventional lower extremity strength training exercises (such as squats) did not improve paretic limb strength, suggesting the patient may have relied on compensatory strategies using the non-paretic side.

Next, the reduction in fall incidents observed during Phase B may be attributed to the DBT intervention improving balance control. A previous study has demonstrated that intensive lower limb constraint-induced therapy (CI therapy), which promotes the use of the paretic side, can positively influence balance function [14]. The DBT intervention requires active engagement of both the paretic and non-paretic sides during lateral movements, fostering more challenging balance control compared to regular gait training. This bilateral engagement likely enhanced dynamic stability, contributing to fall prevention. Key elements included multi-directional stepping and frequent lower limb movements during lane transition, which incorporate essential fall-prevention skills such as push-off strength and perturbation control. Additionally, as knee extension strength is closely associated with balance ability, the synergistic effect of increased paretic limb strength and improved dynamic balance control likely played a significant role in reducing falls [15].

Finally, while detailed validation through questionnaires was not conducted, the patient reported increased confidence in outdoor walking and reduced fear of falling, as reflected in the improvement in the mobility item of the EQ5D. The DBT incorporated repeated multidirectional stepping with randomly displayed numbers, simulating unpredictable environments and providing dynamic balance training that likely restored confidence in outdoor mobility. Improved balance and reduced fear of falling may also enhance social participation. However, as social participation was not directly measured in this case report, future studies are needed to investigate the relationship between these improvements and social participation outcomes.

This case report had several limitations. It was based on a retrospective comparison of Phase A and Phase B interventions; thus, the reported improvements in paretic knee extension strength, reduction in falls, and enhanced QOL following DBT implementation may not be generalizable to other individuals with chronic stroke. Studies with larger samples are required to confirm the efficacy of DBT in stroke rehabilitation. Additionally, the absence of a baseline balance assessment limits the reported associations. However, specific improvements were noted after the DBT intervention, particularly in some sub-items of the BBS, such as tandem stance and single leg standing. While the total BBS score change did not exceed the minimal detectable change of 2.7, these gains suggest positive effects on higher-level balance tasks [16]. Although the patient was independent in walking and activities of daily living, her balance ability was below the fall risk cutoff of 46.5 on the BBS [17]. Although walking speed improved during Phase A, recurrent falls indicate a reliance on compensatory strategies using the non-paretic side. In contrast, the introduction of DBT in Phase B promoted active engagement of the paretic limb, contributing to a BBS score approaching the fall risk threshold and a reduction in falls. Regarding walking speed, a slight decrease of 0.01 m/s was observed after Phase B. However, a previous study reported that the minimal detectable change for 10 m walking speed in chronic stroke patients with baseline speeds of 0.49-0.93 m/s was 0.15 m/s [18]. Since DBT was not designed as fast walking practice, the minimal change in walking speed cannot be explained. Nonetheless, repetitive stepping exercises emphasized during DBT likely increased paretic muscle strength, reduced fall frequency, and alleviated the fear of falling, reflecting task-specific adaptations to the DBT training.

## Conclusions

In this case report, we implemented a DBT intervention for a chronic stroke patient who had experienced an increasing number of falls. The intervention successfully reduced the occurrence of falls during the intervention period. These findings suggest that DBT may have specific utility not only for elderly individuals but also for stroke patients who have independent walking ability yet impaired balance.
